# Structural basis of IRGB10 oligomerization by GTP hydrolysis

**DOI:** 10.3389/fimmu.2023.1254415

**Published:** 2023-08-29

**Authors:** Hyun Ji Ha, Ju Hyeong Kim, Gwan Hee Lee, Subin Kim, Hyun Ho Park

**Affiliations:** ^1^ College of Pharmacy, Chung-Ang University, Seoul, Republic of Korea; ^2^ Department of Global Innovative Drugs, Graduate School of Chung-Ang University, Seoul, Republic of Korea

**Keywords:** crystal structure, GTPase, innate immunity, IRG proteins, oligomerization

## Abstract

Immunity-related GTPase B10 (IRGB10) is a crucial member of the interferon (IFN)-inducible GTPases and plays a vital role in host defense mechanisms. Following infection, IRGB10 is induced by IFNs and functions by liberating pathogenic ligands to activate the inflammasome through direct disruption of the pathogen membrane. Despite extensive investigation into the significance of the cell-autonomous immune response, the precise molecular mechanism underlying IRGB10–mediated microbial membrane disruption remains elusive. Herein, we present two structures of different forms of IRGB10, the nucleotide-free and GppNHp-bound forms. Based on these structures, we identified that IRGB10 exists as a monomer in nucleotide-free and GTP binding states. Additionally, we identified that GTP hydrolysis is critical for dimer formation and further oligomerization of IRGB10. Building upon these observations, we propose a mechanistic model to elucidate the working mechanism of IRGB10 during pathogen membrane disruption.

## 1 Introduction

Pathogen invasion triggers various immune responses in living organisms, the production of interferon through the immune response representing one such example (1, 2). The produced interferon triggers an intracellular signal to induce immune response-related gene expression, and the resulting proteins then contribute to proper host defense through various ways (3–5). In the event that these defense processes fail, various diseases, including immunodeficiency, can occur (6, 7). Interferon (IFN)-inducible GTPase is one of the main immune response-related proteins induced by interferons. IFN-inducible GTPases are characterized by the ability to protect the host by eliminating pathogens using their GTPase activity (8, 9). The GTPase family is divided into four groups, including Mx GTPase, very large inducible GTPase (VLIG), guanylate-binding protein (GBP), and immunity-related GTPase (IRG), according to the type of inducer interferon and physical-molecular mass of the proteins (8, 10–12). Type 1 interferons (alpha and beta) induce Mx GTPases (72–82 kDa) (13), while type 2 interferon (gamma) induces VLIG (molecular weight: 200–285 kDa) (14), GBP (molecular weight: 65–73 kDa) (15), and IRG (molecular weight: 21–47 kDa) (16) GTPases.

The IRG family, also called p47 GTPases, comprise IFN-inducible GTPases, which are involved in the early immune response. In mice, a total of 23 genes (*IRGA 1–8, IRGB 1–10, IRGC, IRGD, IRGM 1–3*) have been identified as IRG family, while only a single full-length IRGC and truncated IRGM have been identified as human IRG family (8, 17). Similar to other GTPases, the IRG family possesses a GTPase domain containing a highly conserved P-loop to which GTP binds. The IRG family is divided into two classes, the GKS class and GMS class, according to the P-loop sequence (18). The IRG family GKS class contains a conserved G-x(4)-GKS pattern in the P-loop, while the GMS class contains a G-x(4)-GMS sequence pattern in the P-loop. All IRG families except IRGM (GMS class) are included in the GKS class (17, 18). The IRG family is known to contribute to cell-autonomous immune responses against invasion by various pathogens (19, 20).

Although their detailed working mechanisms are unclear, several studies on IRGB10, an IRG family member, have indicated that the IRG family mediates pathogen membrane disruption in collaboration with the GBP family, which is critical for the host defense mechanism (20). During this pathogen membrane disruption stage, pathogenic products, such as DNA and lipopolysaccharide (LPS), are released from the pathogen and induce the formation of inflammasomes to further promote the host immune response (20). In the case of IRGA6 and IRGB6, IRGA6 directly binds to the pathogen membrane using N-terminal myristoylation, whereas IRGB6 is not involved in the membrane disruption. However, it remains unclear whether other IRG family proteins can also directly interact with pathogens and contribute to pathogen membrane disruption similar to IRGB10 and IRGA6 (20–24). The various IRG families may have their own action mechanism for the immune system.

Among the IRG family, the structures of IRGA6 (25), IRGB6 (24), and IRGB10 (26) have been elucidated, with several studies revealing that they share similar structures, comprising two distinct domains, a helical domain, and a GTPase domain. The IRG family usually forms a unique head-to-head dimer, as well as a further oligomer during pathogen membrane disruption (26, 27). To form head-to-head dimers, IRGA6 uses the P-loop and switch I region of the GTPase domain, whereas IRGB10 uses one of the helices of the GTPase domain (26, 27). Without clear experimental data, we previously suggested a structural model of pathogen membrane disruption by IRGB10 using the elucidated GDP-bound dimeric IRGB10 structure (26). Additionally, we speculated that the structure of IRGB10 is altered by GTP hydrolysis similar to that of other GTPase proteins, such as Atlastin1, which is structurally related to the IRG families. We also speculated that GTP hydrolysis and the presence or absence of nucleotides impact the function of IRGB10. Although these assumptions were made based on the GDP-bound structure of IRGB10 in our previous study, several unanswered questions remain regarding the functional mechanism of IRGB10. First, how does nucleotide binding affect the structure and function of IRGB10? Second, is GTP hydrolysis critical for the oligomerization of IRGB10? Lastly, how can IRGB10 make pores in the pathogen membrane? To answer these questions, in this study, we elucidated two more IRGB10 structures, including nucleotide-free and GppNHp-bound forms. Additionally, we reveal that GTP hydrolysis is critical for dimer formation and further oligomerization of IRGB10. Based on the current structural, biochemical, and biophysical studies, we provide a model of IRGB10-mediated pore formation on pathogen membranes in a step-by-step manner.

## 2 Methods

### 2.1 Expression and purification of GDP-bound IRGB10

The purification details of GDP-bound IRGB10 were introduced in a previous study (26). Briefly, the plasmid containing the *IRGB10* gene was transformed into *Escherichia coli* BL21 (DE3) competent cells. Subsequently, the cells were coated onto plates containing Luria-Bertani (LB) agar and incubated overnight at 37°C. A single colony was inoculated into 5–10 mL of LB medium, transferred to 1 L of LB medium, and incubated at 37°C until the optical density (OD) reached ~0.7. Subsequently, 0.5 mM isopropyl β-D-thiogalactopyranoside was added to the medium to induce protein expression, and the cells were incubated overnight at 20°C. After overnight incubation, cells expressing IRGB10 were collected by centrifugation and suspended in 16 mL of lysis buffer (20 mM Tris-HCl pH 8.0, 500 mM NaCl, and 5 mM imidazole). Subsequently, the cells were disrupted by sonication on ice. The cell lysates were centrifuged at 10,000 g for 30 min at 4°C to remove the cell debris, and the supernatant was incubated with nickel-nitrilotriacetic acid (Ni-NTA) affinity resin (Qiagen, Hilden, Germany). After incubation, the supernatant was loaded onto a gravity-flow column (Bio-Rad, Hercules, CA, USA) and the resin was washed with 50 mL of washing buffer (20 mM Tris-HCl pH 8.0, 500 mM NaCl, and 25 mM imidazole) to remove impurities. The target protein was eluted from the resin in the column using elution buffer (20 mM Tris-HCl pH 8.0, 500 mM NaCl, and 250 mM imidazole). The eluted protein was further purified with size-exclusion chromatography (SEC) using SEC buffer (20 mM Tris-HCl pH 8.0, and 150 mM NaCl). The target protein was eluted at around 13 mL, concentrated to 10–12 mg/mL, and stored for structural and biochemical studies.

### 2.2 Expression and purification of nucleotide-free IRGB10

The same IRGB10 expression clone that was used for the expression and purification of GDP-bound IRGB10 was used for the expression and purification of nucleotide-free IRGB10. The expression in *E. coli* and affinity chromatography was performed using the same method as that used for the purification of GDP-bound IRGB10. During the washing step, the resin was washed with 30 mL of the first washing buffer (20 mM Tris-HCl pH 8.0, 500 mM NaCl), before transferring the washed Ni-NTA resin to 50 mL of the second washing buffer (20 mM Tris-HCl pH 8.0, 1.5 M NaCl) and incubating for 30 min at room temperature. Subsequently, the incubated Ni-NTA resin was reloaded into a gravity column and washed again with 30 mL of the third washing buffer (20 mM Tris-HCl pH 8.0, 500 mM NaCl, 25 mM imidazole). The target protein was eluted using 3 mL elution buffer applied onto the column, and the eluted proteins were loaded onto the SEC column. A Superdex 200 Increase 10/300 GL column (GE Healthcare, Chicago, USA), which had been pre-equilibrated with the SEC buffer, was used in the SEC experiment. The absence of nucleotides was checked by UV absorbance (A260/A280), as outlined in a previous study (28).

### 2.3 Multi-angle light scattering

The molar masses of nucleotide-free IRGB10, GppNHp-bound IRGB10, and K81A mutant IRGB10 were determined by multi-angle light scattering (MALS). The purified target protein was injected into a Superdex 200 HR 10/30 gel-filtration column (GE Healthcare) that had been pre-equilibrated in buffer containing 20 mM Tris-HCl pH 8.0 and 150 mM NaCl. The chromatography system was coupled to a MALS detector (mini-DAWN TREOS) and a refractive index detector (Optilab DSP) (Wyatt Technology). The data were collected every 0.5 s at a flow rate of 0.4 mL/min and then analyzed using the ASTRA program.

### 2.4 Crystallization and data collection

Crystallization of nucleotide-free IRGB10 was performed at 20°C using the hanging drop vapor diffusion method. Initial crystals were screened using a crystallization screening kit from molecular Dimensions, Hampton Research. The crystals were grown on plates by equilibrating a mixed drop of 1 μL protein solution (8–9 mg/mL protein in SEC buffer) and 1 μL reservoir solution containing 0.1 M Tris-HCl pH 7.0, 2.0 M (NH_4_)_2_SO_4,_ and 0.2 M Li_2_SO_4_ against 0.3 mL reservoir solution. The crystallization conditions were further optimized by experimenting with various concentrations and pH values of (NH_4_)_2_SO_4_. The optimized crystals appeared in the presence of 0.1 M Tris-HCl pH 7.2, 1.8 M (NH_4_)_2_SO_4_, and 0.2 M Li_2_SO_4_.

Crystallization of the GppNHp-bound IRGB10 was performed at 20°C using the hanging drop vapor diffusion method. Just before crystallization, 10 mM GppNHp and 2 mM MgCl_2_ were added to 11 mg/mL nucleotide-free IRGB10 protein sample and incubated for 20 min. After incubation, the mixture was screened using a crystallization screening kit. Initial crystals were grown on a reservoir solution containing 0.1 M Tris-HCl pH 8.5, 20% (w/v) polyethylene glycol (PEG), 2,000 monomethyl ether (MME), and 0.2 M trimethylamine n-oxide. The diffraction data sets were collected at the BL-5C beamline of Pohang Accelerator Laboratory (PAL) (Pohang, Republic of Korea). Data processing and scaling were conducted using the HKL2000 package.

### 2.5 Structure determination and analysis

The structures of nucleotide-free and GppNHp-bound IRGB10 were determined by the molecular-replacement (MR) phasing method using the Phaser program in the PHENIX program (29). The previously solved IRGB10 GDP-bound structure (PDB ID: 7C3K) was used as the search model. Model building and refinement were conducted by COOT (30) and Refmac5 (31), respectively. Water molecules were added using the ARP/wARP function in Refmac5. The geometry was inspected using PROCHECK and was found to be acceptable. The quality of the model was confirmed using MolProbity (32). All structure figures were created using PyMOL (33).

### 2.6 Oligomerization measurement

Oligomerization of IRGB10 was assessed using turbidity measurement (34, 35). Assembly of the IRGB10 oligomer was determined by measuring the absorbance at 350 nm UV using a Nanophotometer NP80 (IMPLEN, Munich, Germany) at 37°C. Purified proteins were concentrated to 100 μM ~ 500 μM and placed in quartz cuvettes. The protein only was placed in cuvettes before starting the measurement. After 500 s, 10 μL of the GTP and MgCl_2_ mixture was added. After finishing the measurement, the protein samples were centrifuged at 10,000 g for 10 min at 4°C to remove aggregates. The remaining solution was loaded onto a SEC column, which had been pre-equilibrated with buffer containing 20 mM Tris-HCl pH 8.0, 150 mM to determine the dimer form of IRGB10.

### 2.7 Native-PAGE

Self-oligomerization of IRGB10 due to GTPase activity was monitored by native-PAGE using a Phast system (GE Healthcare). Pre-made 8%–25% acrylamide gradient gels (GE Healthcare) were used for this experiment. The shifted bands on the gel were stained with Coomassie Brilliant Blue. Purified nucleotide-free IRGB10 was mixed and incubated with different concentrations of GTP and MgCl_2_ mixtures at 37°C for 30 min, before loading the mixture onto native gels.

### 2.8 Circular dichroism measurements

A tentative structural change of IRGB10 caused by GTPase activity was detected using CD measurements. A J-1500 spectropolarimeter at the Korea Basic Science Institute (Osong, South Korea) was used for the CD experiment. The spectra were obtained from 200 to 260 nm at 25°C in a 1-mm pathlength quartz cuvette using a bandwidth of 1.0 nm, a 100 nm/min speed, and a 5-s response time. Three scans were accumulated and averaged. The concentration of nucleotide-free IRGB10 and K81A mutant IRGB10 in the SEC buffer was 0.3–0.4 mg/mL. Next, 2 mM GTP and 0.2 mM MgCl_2_ mixture was added to the protein to generate a nucleotide-free IRGB10 + GTP sample. The mixture was incubated at 25°C for 30 min just before injecting the sample into the spectropolarimeter.

### 2.9 Accession codes

The atomic coordinates and structure factors of nucleotide-free and GppNHp-bound IRGB10 were deposited in the Protein Data bank under accession numbers 8JQY and 8JQZ, respectively.

## 3 Results

### 3.1 Nucleotide-free IRGB10 is a monomer in solution

**Figure 1:**
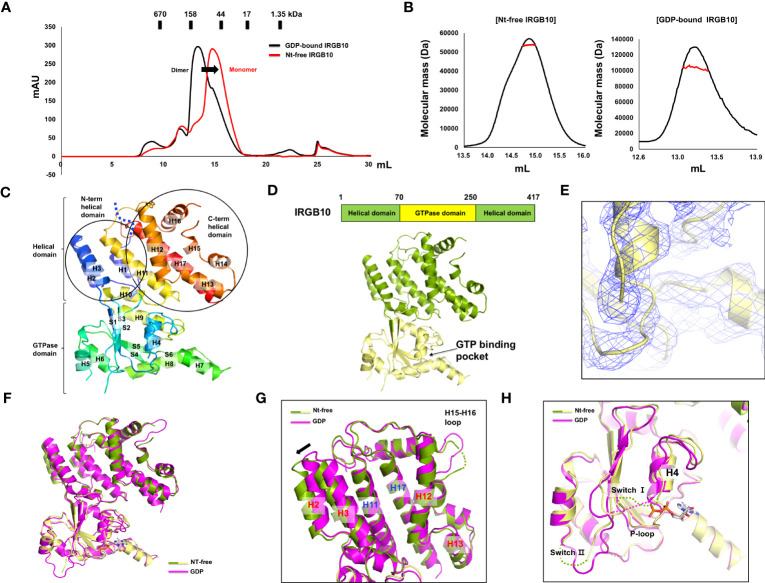
Structure of nucleotide-free IRGB10. **(A)** Profiles of the size-exclusion chromatography (SEC) of GDP-bound IRGB10 (black line) and nucleotide-free IRGB10 (red-line). The shifted peak is indicated by the black arrow. **(B)** Multi-angle light scattering (MALS) profiles derived from the SEC peak from the nucleotide-free IRGB10 (left panel) and GDP-bound IRGB10 (right panel). Red line indicates the experimental molecular mass. **(C)** Overall structure of nucleotide-free IRGB10. The rainbow-colored cartoon representation of monomeric nucleotide-free IRGB10 is shown. The chain from the N- to C-terminus is colored blue to red. Helices and sheets are labeled with H and S, respectively. The missing N-terminal loop in indicated by the blue dotted line. **(D)** The domain boundary and overall structure of IRGB10. The relative positions of the helical domain and the GTPase domains are shown in the bar diagram at the top. **(E)** Close-up view of the nucleotide binding pocket in the GTPase domain of IRGB10. The *2Fo-Fc* electron density map contoured at the 1σ level is indicated by the blue mesh. **(F)** Structural comparison of nucleotide-free IRGB10 (mixed green and yellow) with GDP-bound IRGB10 (magenta) by structural superposition. **(G)** Close-up view of the helical domains from panel **(F)** The structurally misaligned region is indicated by the black arrow. **(H)** Close-up view of the GTPase domain from panel **(F)** Missing, unconstructed loops in the model are indicated by dotted lines.

Many GTPases, including the GTPase domain-containing dynamin family, function appropriately by altering their structure and stoichiometry dependent on their GTP/GDP binding state and GTPase activity (36, 37). To reveal the accurate working mechanism of IRGB10 in the process of pathogen membrane disruption, whose function might be dependent on the state of nucleotide binding and hydrolysis capacity, we attempted to solve the structures of the nucleotide-free IRGB10 and IRGB10/GTP complexes. As we observed that endogenous GDP in *E. coli* was automatically incorporated into IRGB10 during the purification step, we used an additional high-salt washing step during an affinity chromatography step, which has been used previously to remove nucleotides from binding proteins, to obtain nucleotide-free IRGB10 (38). The absence of nucleotides was checked by UV absorbance (A260/A280), as has been outlined previously (28). This experiment showed that the absorbance value of nucleotide-free IRGB10 was 0.67~0.64, while that for GDP-bound IRGB10 was 1.02~1.36, indicating that GDP was washed out during the purification step (**Supplementary Figure 1**). Next, purified nucleotide-free IRGB10 was applied to SEC, with a GDP-bound IRGB10 sample used for size control. Comparison of the SEC profiles indicated that the main elution peak of nucleotide-free IRGB10 was moved to the monomer size position, although the overall generated peaks on the SEC profiles were similar (**Figure 1A**). The molecular size of nucleotide-free IRGB10 was accurately determined by MALS, which was then used to calculate the absolute molecular mass of the protein particle. The results of MALS showed that the molecular weight of the tentative monomeric peak from nucleotide-free IRGB10 was 53.65 kDa (± 0.7%), whereas the molecular mass of the dimeric GDP-bound IRGB10 was 102.72 (± 1.8%) (**Figure 1B**). These results indicated that the dimeric GDP-bound IRGB10 became monomeric when IRGB10 lost its GDP. Purified nucleotide-free IRGB10 was successfully crystallized, which allowed the structure of the monomeric nucleotide-free IRGB10 to be solved. The crystallographic data and refinement statistics are summarized in **Table 1**. Unlike dimeric GDP-bound IRGB10, a single molecule of IRGB10 was detected in the crystallographic asymmetric unit (ASU). The overall structure of monomeric nucleotide-free IRGB10 was almost identical to that of GDP-bound IRGB10, which was composed of a helical domain formed by N-terminal, C-terminal, and GTPase domains, which is a typical domain composition of the IRG family (**Figures 1C, D**). The GTPase domain consisted of six β-sheets (S1–S6) and six α-helices (H4–H9), while the helical domain consisted of eleven α-helices, including H1~H3 from the N-terminus region and H10~H17 from the C-terminus region. The model of nucleotide-free IRGB10 was constructed from residue 16 to residue 406. The LEH residues at the C-terminus, which were from the plasmid construct, were included in the final model. The electron density of the N-terminus residues and several loops, including switches I and II in the GTPase domain, were not visible in the model (**Figure 1C**). These parts of the structure could not be constructed due to poor electron density. The unconstructed N-terminus and several loops in the GTPase domains around these structures were also observed in structural studies of IRGA6 and dimeric GDP-bound IRGB10; this indicates that the N-terminus loop, containing around 13–15 residues, and several loops, including switches I and II at the GTPase domain, are extremely flexible and unstructured regions (25). As we removed GDP from IRGB10 during the purification step and found that nucleotide-free IRGB10 became a monomer in solution, using the current structure, we first sought to investigate whether GDP or GTP is in the GTPase domain. The electron density search revealed no traceable electron density for GDP in the typical nucleotide-binding site of the GTPase domain (**Figure 1E**).

**Table 1:** Data collection and refinement statistics.

	Nucleotide-free	GppNHp-bound
Data collection		
Space group	*P 3_2_ 2 1*	*P 1 2_1_ 1*
Unit cell parameter		
*a*, *b*, *c* (Å) *α*, *β*, *γ* (°) Resolution range (Å) Total reflections Unique reflections Multiplicity^1^ Completeness (%)^1^ Mean *I*/σ (*I*)^1^ *R*_merge_ (%)^1,2^ Wilson *B*-factor (Å^2^)	190.24, 190.24, 38.88 90, 90, 120 29.59–3.68 184231 8893 20.7 (21.9) 99.66 (100.00) 18.54 (1.84) 18.04 (215.1) 138.93	62.64, 62.44, 119.23 90, 99.52, 90 29.43–3.05 116108 17524 6.6 (6.8) 99.44 (99.43) 12.86 (1.80) 13.13(111.3) 84.19
Refinement		
Resolution range (Å) Reflections *R*_work_ (%)^1^ *R*_free_ (%)^1^ No. of molecules in ASU^3^ No. of non-hydrogen atoms Macromolecules	29.59–3.68 8892 24.94 (34.29) 27.19 (36.96) 1 3032 3032	29.43–3.05 17494 23.19 (35.46) 26.50 (35.74) 2 6137 6073
Ligands		64
Average *B*-factor values (Å^2^) Macromolecules	155.45 155.45	93.03 92.35
Ligands		158.07
Ramachandran plot:		
favored/allowed/outliers (%) Rotamer outliers (%) Clashscore RMSD bonds (Å)/angles (°)	97.26/2.74/0.00 0.00 11.35 0.003/0.58	98.77/1.23/0.00 0.00 5.54 0.003/0.92

^1^ Values for the outermost resolution shell are shown in parentheses.

^2^ R_merge_ = Σ_h_ Σ_i_ |I(h)_i_ − <I(h)>|/Σ_h_ Σ_i_ I(h)_i_, where I(h) is the observed intensity of reflection h, and <I(h)> is the average intensity obtained from multiple measurements.

^3^ Crystallographic Asymmetric Unit.

Next, we compared the structure of the nucleotide-free IRGB10 to that of the GDP-bound IRGB10 (PDB ID: 7C3K) to analyze any structural changes that might occur by the loss of nucleotides in IRGB10. Pair-wise structural alignments between nucleotide-free and GDP-bound IRGB10 showed that the overall structures were similar to each other, with a RMSD between the two structures of 1.3 Å (**Figure 1F**). However, close-up analysis showed that the H2 and H3 helical domains formed by the N-terminal part of IRGB10 were dislocated from the positions of the H2 and H3 regions of GDP-bound IRGB10 (**Figure 1G**). In contrast, the last part of the helical domain that was constructed by the C-terminal part of IRGB10 was identical to that in GDP-bound IRGB10, indicating that binding nucleotide or GTP hydrolysis causes a slight structural alteration of IRGB10. Although the structures of the GTPase domain of each structure were almost identical, the positions of several loops were not. The structures of switches I and II, both of which are critical for GTPase activity, were unconstructed in nucleotide-free IRGB10, while only switch I was unconstructed in GDP-bound IRGB10 (**Figure 1H**). Interestingly, the P-loop, which is critical for nucleotide binding and GTP hydrolysis, was well constructed in both structures, indicating that the formation and location of the proper positioning of the P-loop is independent of nucleotide binding, contrary to what we have argued in a previous structural study of GDP-bound IRGB10 (26).

### 3.2 GTP hydrolysis causes dimerization and further oligomerization of IRGB10

**Figure 2:**
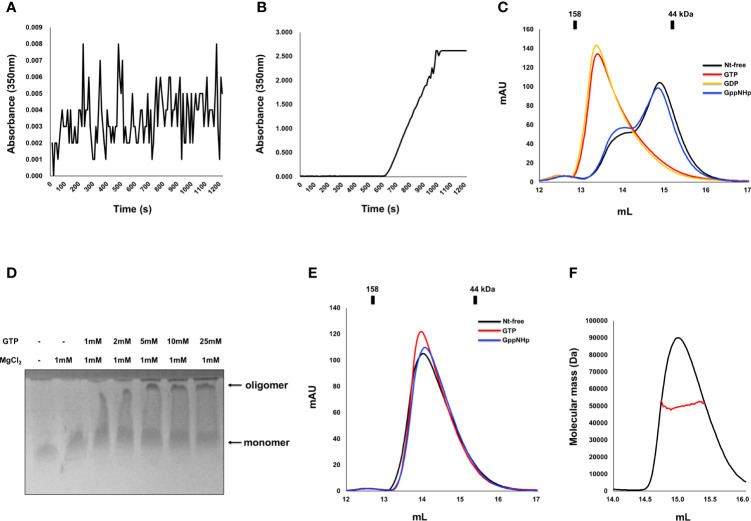
Dimerization and further oligomerization of IRGB10 by GTP hydrolysis. **(A, B)** Assembly of the IRGB10 oligomer as measured by turbidity changes. Turbidity changes of solutions containing nucleotide-free IRGB10 were measured upon addition of water for control **(A)** and GTP/MgCl_2_ **(B)**. **(C)** SEC profiles of nucleotide-free (Nt-free) IRGB10 (black line), GTP-added IRGB10 (red line), GDP-added IRGB10 (yellow line), and GppNHp-added IRGB10 (blue line). **(D)** Native-PAGE of IRGB10 incubated with various concentrations of GTP in the presence or absence of MgCl_2_. The concentrations of GTP incubated with IRGB10 are indicated. **(E)** SEC profiles of K81A mutant IRGB10. **(F)** MALS profile derived from the SEC peak from the K81A mutant IRGB10. Red line indicates the experimental molecular mass.

Indeed, as we observed that nucleotide binding affects the stoichiometry change of IRGB10, we also investigated the effect of GTPase activity and GTP binding on any oligomeric and structural changes of IRGB10. To accomplish this, we first performed a turbidity assay that has been used previously to analyze the oligomerization of IRGA6 (34). The oligomerization of IRGB10 was detected by checking the absorbance of 350 nm UV light. After adding the GTP/MgCl_2_ mixture to GDP-bound IRGB10, UV absorbance was not detected for 1200 s (**Figure 2A**). However, when the GTP/MgCl_2_ mixture was added to nucleotide-free IRGB10, a considerable increase in UV absorbance was detected 600 s after GTP addition (**Figure 2B**). This UV absorbance was not detected when a non-hydrolysable GTP analog (GppNHp) was supplied to nucleotide-free IRGB10 (**Supplementary Figure 2**). The results of these turbidity assays indicated that GTP hydrolysis caused the oligomerization of IRGB10. Moreover, visible IRGB10 oligomeric particles were detected in the tube containing nucleotide-free IRGB10 following GTP addition. After removing those oligomeric particles by centrifugation, the solution was loaded onto SEC to determine the remnants in the solution. As GTPase hydrolyzes GTP to GDP, we speculated that the dimeric form of GDP-bound IRGB10 would be observed if the hydrolyzed product of GDP was incorporated into IRGB10 after hydrolysis. As expected, the SEC profile showed that the last portion of IRGB10 after GTP hydrolysis was a dimeric size and was eluted around the 13–14 position where dimeric GDP-bound IRGB10 was eluted (**Figure 2C**). When the non-hydrolysable GTP analog GppNHp was added to nucleotide-free IRGB10, no oligomeric particles were observed in the tube and the SEC profile showed a monomeric size (**Figure 2C**), indicating that GTP hydrolysis is critical for the dimerization and further oligomerization of IRGB10. The effect of GDP addition on the nt-free IRGB10 was also assessed by performing the same turbidity (**Supplementary Figure 3**). As shown at the **S3 Figure**, GDP addition did not produce oligomeric peak although a little absorbance was detected at 200 sec point after addition of GDP/MgCl_2_. This indicated that GTP hydrolysis is critical step for IRGB10 oligomerization. The GTP hydrolysis-mediated oligomerization of IRGB10 was confirmed by native PAGE, which is another oligomerization detection assay. As shown in **Figure 2D**, the newly formed oligomeric band was detected by the addition of GTP, indicating that GTP addition caused IRGB10 oligomerization, which was observed in the turbidity assay. Finally, we attempted to confirm whether GTP hydrolysis of IRGB10 is essential for dimer formation and further oligomer formation by constructing mutants that cannot hydrolyze GTP. To perform this experiment, K81, a catalytically important residue identified from previous study (19), was mutated to alanine to produce the K81A mutant, which is the GTP-locked form of IRGB10. Using this GTP-locked form of IRGB10, we performed a turbidity assay and SEC-MALS. Unlike wildtype IRGB10, K81A did not produce visible oligomeric particles following the addition of GTP. Additionally, K81A did not produce a dimeric peak on the SEC profile when GTP was added to K81A (**Figure 2E**). All three SEC samples, including nucleotide-free K81A, K81A with GTP, and K81A with GppNHp, were eluted at the monomer position at SEC-MALS (**Figures 2E, F**). In addition, K81A did not produced dimeric peak in the presence of GDP (**Supplementary Figure 4**). These additional experiments confirmed that GTP hydrolysis is essential for dimer formation and further oligomerization of IRGB10, which may be critical for pathogen membrane disruption. Finally, we elucidated the role of the dimer PPI of IRGB10 on the oligomerization of IRGB10. To evaluate this, we used dimer PPI disrupting mutant D185R, which has been identified by our previous study as PPI interfering mutant. The turbidity assay showed that nt-free D185R mutant failed to produce oligomeric peak after addition of GTP/MgCl_2_ (**Supplementary Figure 5**). Based on this experiment, we concluded that dimerization is a seed of further oligomerization of IRGB10.

### 3.3 GppNHp-bound IRGB10 is a monomer in solution

**Figure 3:**
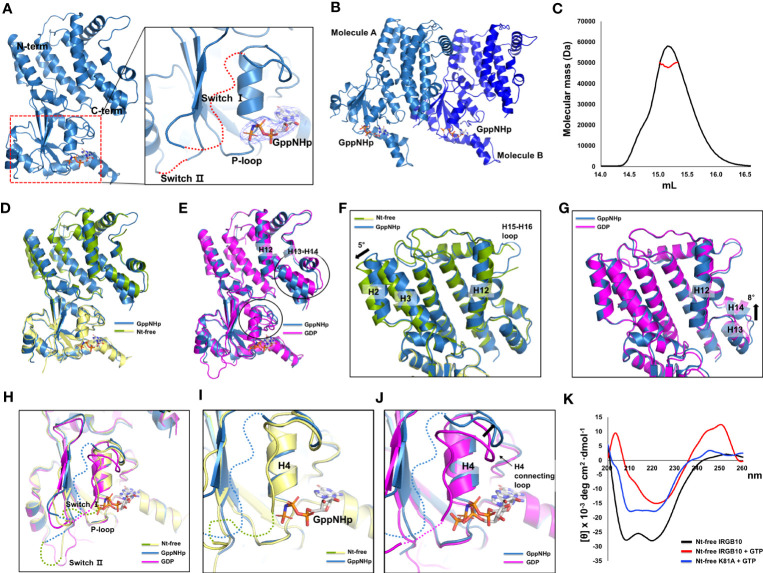
Structure of GppNHp-bound IRGB10. **(A)** Overall structure of GppNHp-bound IRGB10. Close-up view of the nucleotide binding pocket in the GTPase domain of IRGB10 shown in the right panel. The missing unconstructed switch I and II loops are indicated by red dotted lines. The *2Fo-Fc* electron density map contoured at the 1σ level around GppNHp is indicated by blue mesh. **(B)** A cartoon representation of two GppNHp-bound IRGB10s presented in an asymmetric unit. **(C)** Multi-angle light scattering (MALS) profiles derived from the SEC peak from GppNHp-bound IRGB10. Red line indicates the experimental molecular mass. **(D)** Structural comparison of GppNHp-bound IRGB10 (metal blue) with nucleotide-free IRGB10 (mixed green and yellow) by structural superposition. **(E)** Structural comparison of GppNHp-bound IRGB10 (metal blue) with GDP-bound IRGB10 (magenta) by structural superposition. Two structurally misaligned regions are indicated by black circles. **(F)** Close-up view of the helical domains from panel **(D)**. The structurally misaligned region is indicated by a black arrow. **(G)** Close-up view of the helical domains from panel **(E)**. The structurally misaligned region is indicated by a black arrow. **(H)** Structural comparison of the GTPase domains of GppNHp-bound IRGB10 (metal blue) with GDP-bound IRGB10 (magenta) and nucleotide-free IRGB10 (mixed green and yellow) by structural superposition. **(I)** Close-up view of the nucleotide pocket from panel H showing GppNHp-bound IRGB10 and nucleotide-free IRGB10. **(J)** Close-up view of the nucleotide pocket from panel H showing GppNHp-bound IRGB10 and GDP-bound IRGB10. The structurally misaligned region and H4 and H4 connecting loop are indicated. **(k)** Circular dichroic spectra of nucleotide-free (Nt-free) IRGB10 (black line), Nt-free IRGB10 provided GTP (red-line), and Nt-free K81A mutant IRGB10 provided GTP (blue line).

The mimetic structure of the GTP-bound form of IRGB10 was also solved using GppNHp, which is a non-hydrolyzable GTP analog. The crystallographic data and refinement statistics are summarized in **Table 1**. The overall structure and numbers of α-helices and β-sheets were similar to previously revealed nucleotide-free and GDP-bound IRGB10 structures. We detected a clear electron density map at the nucleotide-binding site in the GTPase domain responsible for GppNHp (**Figure 3A**). The P-loop was stably fixed in GppNHp-bound IRGB10, while switches I and II remained unstructured (**Figure 3A**). Additionally, the crystallographic asymmetric unit comprised two identical IRGB10 molecules, molecule A and B (**Figure 3B**). As the stoichiometric and structural changes of the IRG family of GTPases are critical for understanding the working mechanism of the IRG family, we next analyzed the stoichiometry of GppNHp-bound IRGB10 in the solution using MALS. The experimentally calculated molecular weight of GppNHp-bound IRGB10 was 48.93 kDa (± 3.941%), indicating that GppNHp-bound IRGB10 is a monomer in the solution (**Figure 3C**), indicating that GTP bound-IRGB10 without GTP hydrolysis is still a monomer in solution.

To comprehend any structural change caused by nucleotide binding and GTPase activity, we next compared the GppNHp structure with nucleotide-free (**Figure 3D**) and GDP-bound (**Figure 3E**) IRGB10 structures by structural superposition analysis. The results of this structural comparison indicated that the overall GppNHp structure is almost identical to that of the nucleotide-free and GDP-bound forms of IRGB10, with RMSDs of 0.8 Å and 1.2 Å, respectively. However, upon closer examination of the helical domain, the locations of several helices were not identical. Indeed, H2 and H3 of the GppNHp-bound form were tilted by approximately 5° compared to those of the nucleotide-free form of IRGB10 (**Figure 3F**). Moreover, compared to the helical domain of the GDP-bound form, H13 and H14 of the GppNHp-bound form were tilted by approximately 8° compared to those of the GDP-bound form of IRGB10 (**Figure 3G**). The largest structural alteration was detected in the nucleotide binding site (**Figure 3H**). Although no structural changes were detected when the GppNHp-bound structure was compared to the nucleotide-free form (**Figure 3I**), distinct movements of the H4 and H4 connecting loops were detected when the GppNHp-bound structure was superposed with the GDP-bound form (**Figure 3J**). Moreover, by conducting a structural comparison of the GTPase domain of the GppNHp-bound IRGB10 with the GDP-bound form, we found that the loops of the GppNHp-bound form were very flexible and unfixed, and the position of H4 connected to the switch I loop in the GDP-bound form was different from that of the GppNHp-bound form (**Figures 3H, J**). All P-loops structures, which are important for nucleotide binding, of the three structures were identical. Finally, we evaluated the far UV circular dichroic (CD) spectra to determine the tentative structural changes of IRGB10 during GTP hydrolysis. The results of this experiment showed that the spectrum patterns were different when nucleotide-free IRGB10 was treated with GTP (**Figure 3K**). The nucleotide-free IRGB10 alone sample produced a typical CD spectrum pattern of α-helical proteins, exhibiting two pronounced minima at 208 nm and 222nm and a maxima at 200 nm. This pattern was not observed when GTP was provided. Moreover, these changes in the CD pattern were not observed when the GTPase activity defect K81A mutant was treated with GTP. In addition, GDP or GppNHp addition also produced a typical CD spectrum pattern produced by wildtype IRGB10 (**Supplementary Figure 6**). These CD experiments indicate that GTP hydrolysis might lead to structural changes in IRGB10.

## 4 Discussion

Given the importance of the field of study and understanding the mechanism underlying membrane disruption, several structures of the IRG family, including IRGA6, IRGB6, and IRGB10, have been revealed so far. Despite this, the functionally important filament-like structures of the IRG family, which are formed for membrane disruption, remain to be elucidated. To better understand the working mechanism of the IRG family, we initially solved the structure of the dimeric GDP-bound form of IRGB10 (26). Although GDP was not included in the protein sample preparation steps, endogenous bacterial GDP was incorporated in the GTPase domain of IRGB10. As the IRG family has a higher affinity for GDP than GTP, the natural production of GDP-bound IRGB10 was not extraordinary (25, 39). We established a method for purification of the nucleotide-free form of IRGB10 and revealed the structures of the nucleotide-free and GppNHp-bound forms of IRGB10 to establish the structural basis of membrane pore formation. Our results showed that IRGB10 existed as a monomer in the nucleotide-free state and became a dimeric form through GTP hydrolysis. During GTPase activation, the GTPase domain was flexible, and several helices underwent structural changes.

Following GTP addition, visible IRGB10 oligomeric particles were detected in the tube containing nucleotide-free IRGB10, which may be aggregates that can be formed due to the absence of membrane. After GTP hydrolysis, IRGB10 is supposed to work on the membrane; however, due to the absence of a membrane or binding partner such as GBP5, oligomeric IRGB10 became aggregated in solution. After removing all of the higher oligomeric particles (or aggregates), the remaining IRGB10 was detected as a dimer in solution, suggesting that the dimeric form is the main functional building block used by the IRG family for membrane disruption of pathogens.

**Figure 4:**
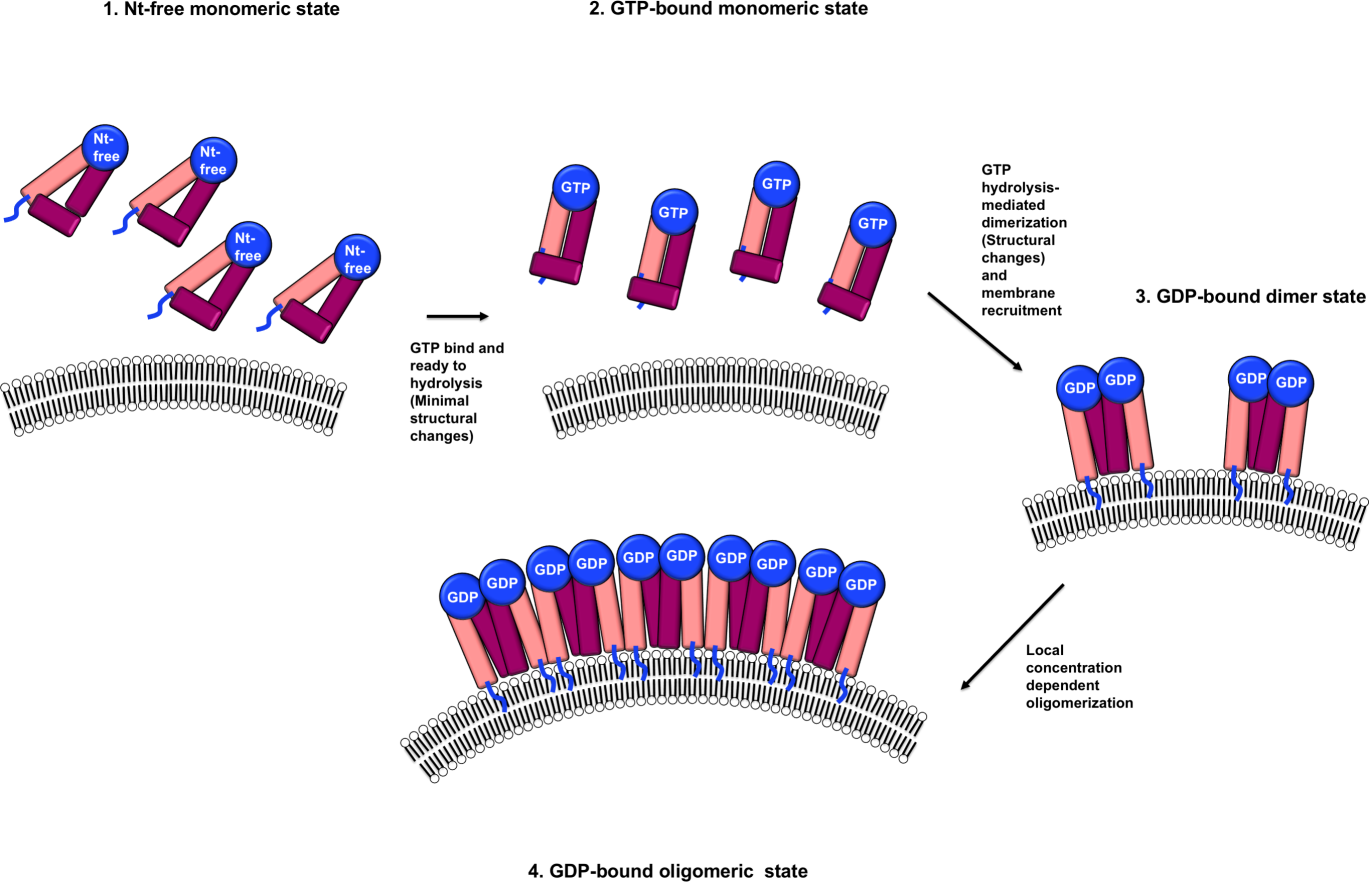
Putative model of a nucleotide and its hydrolysis-mediated membrane pore formation by IRGB10. The blue lines indicate the N-terminus loops where myristoylation occurs.

Structural comparison of the three structures of IRGB10, including nucleotide-free, GDP-bound, and GppNHp-bound, indicated that the structure of the monomeric nucleotide-free form was almost identical to that of the monomeric GppNHp-bound IRGB10. However, the structure of IRGB10 changed if it experienced GTP hydrolysis. Although we observed a limited structural change at both the helical domain and GTPase domain of IRGB10, we expected huge structural changes in the helical domain of IRGB10, which were not observed in our study. In a previous study, although the bacterial dynamin-like protein (BDLP), a member of the IRG-like GTPase dynamin family in bacteria, had a closed conformation in the crystal structures of the nucleotide-free and GDP-bound states (36), this dynamin-like GTPase underwent huge structural changes at the helical domain when GTP was hydrolyzed. This structural change induced by forming the extended helical domain conferred BDLP with the capability to wrap the membrane by further oligomerization in the presence of lipid membrane, as evidence by cryo-EM structure analysis (40). Assuming that IRGB10 works in a manner similar to that of BDLP, GTP hydrolysis-mediated power generation, structural changes to the extended helical domain using generated power, and further oligomerization–mediated membrane disruption may occur, which may be achieved only in the presence of a phospholipid membrane. The possibility of huge stryctyral change of IRGB10 during GTP hydrolysis was indicated by our CD experiments. Although dramatic change of CD profile was detected when IRGB10 was incubated with GTP, this change might be not due to the structural changes but due to the oligomerization of IRGB10 induced by GTP addition. This should be investigated further in near future. Taken together, based on the results of our structural, biochemical, and biophysical studies, we propose a model of IRGB10-mediated pathogen membrane pore formation (**Figure 4**). Initially, IRGB10 without nucleotide forms an inactive monomeric conformation. Once GTP is loaded into the GTPase domain of IRGB10, a minimal structural change, especially at the helical domain, occurs to prepare IRGB10 for action. During the GTP-hydrolysis step, IRGB10 may undergo huge structural changes, which may be critical for the membrane association of IRGB10, dimerization, and further oligomerization for pore formation (**Figure 4**). As we cannot capture the moment at which structural changes of IRGB10 are induced, the types of structural changes that occur during GTP hydrolysis remains an open question.

## Data availability statement

The datasets presented in this study can be found in online repositories. The names of the repository/repositories and accession number(s) can be found in the article/**Supplementary Material**.

## Author contributions

HH: data curation, investigation, writing – original draft. JK: data curation, writing – original draft. GL: data curation, writing – original draft. SK: data curation, writing – original draft. HP: conceptualization, data curation, funding acquisition, project administration, supervision, writing – review & editing, writing – original draft.
