# Supplementary material for: Structural basis of IRGB10 oligomerization by GTP hydrolysis
Source: Front Immunol. 2023 Aug 29;14:1254415. doi: 10.3389/fimmu.2023.1254415 (PMC10495984; doi:10.3389/fimmu.2023.1254415)
Supplement: Supplementary file 1 [file DataSheet_1.docx]

Structural basis of IRGB10 oligomerization by GTP hydrolysis

**Hyun Ji Ha^1^, Ju Hyeong Kim^1,2^, Gwan Hee Lee^1,2^, Subin Kim^1,2^, and Hyun Ho Park^1,2,*^**

^1^College of Pharmacy, Chung-Ang University, Seoul, Republic of Korea

^2^Department of Global Innovative Drugs, Graduate School of Chung-Ang University, Seoul 06974, Republic of Korea

*** Correspondence:**Corresponding Author
xrayleox@cau.ac.kr


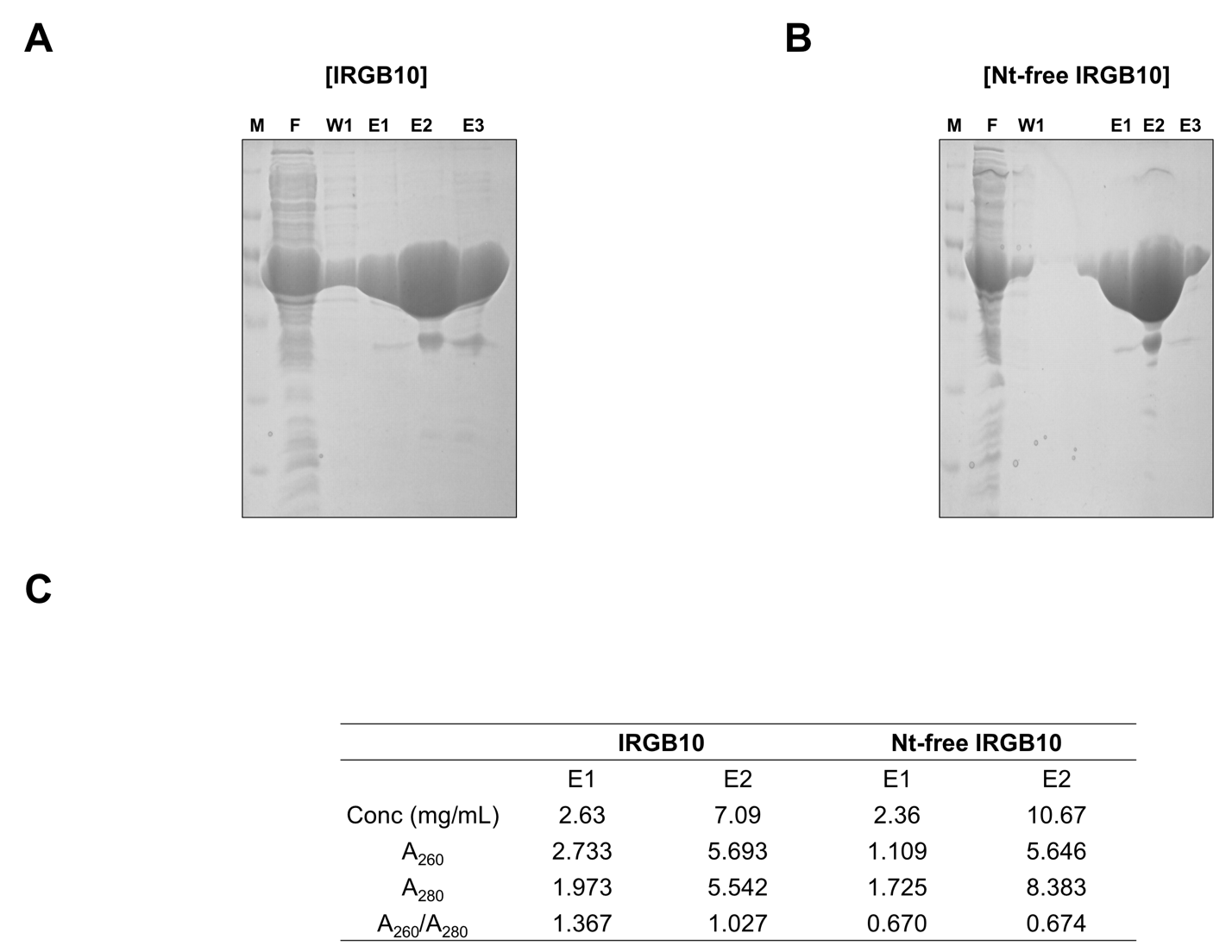


**Supplementary Figure 1**. Purification of nt-free IRGB10 and UV absorbance analsysis for ensuring the absence of nucleotide. A) Ni-NTA affinity purification of GDP-bound IRGB10. SDS-PAGE gel provided. M: size marker, F: flow through, W: washing, E: elution by imidazole. B) Ni-NTA affinity purification of nt-free IRGB10. SDS-PAGE gel provided. C) Table summarizing the UV absorbance.


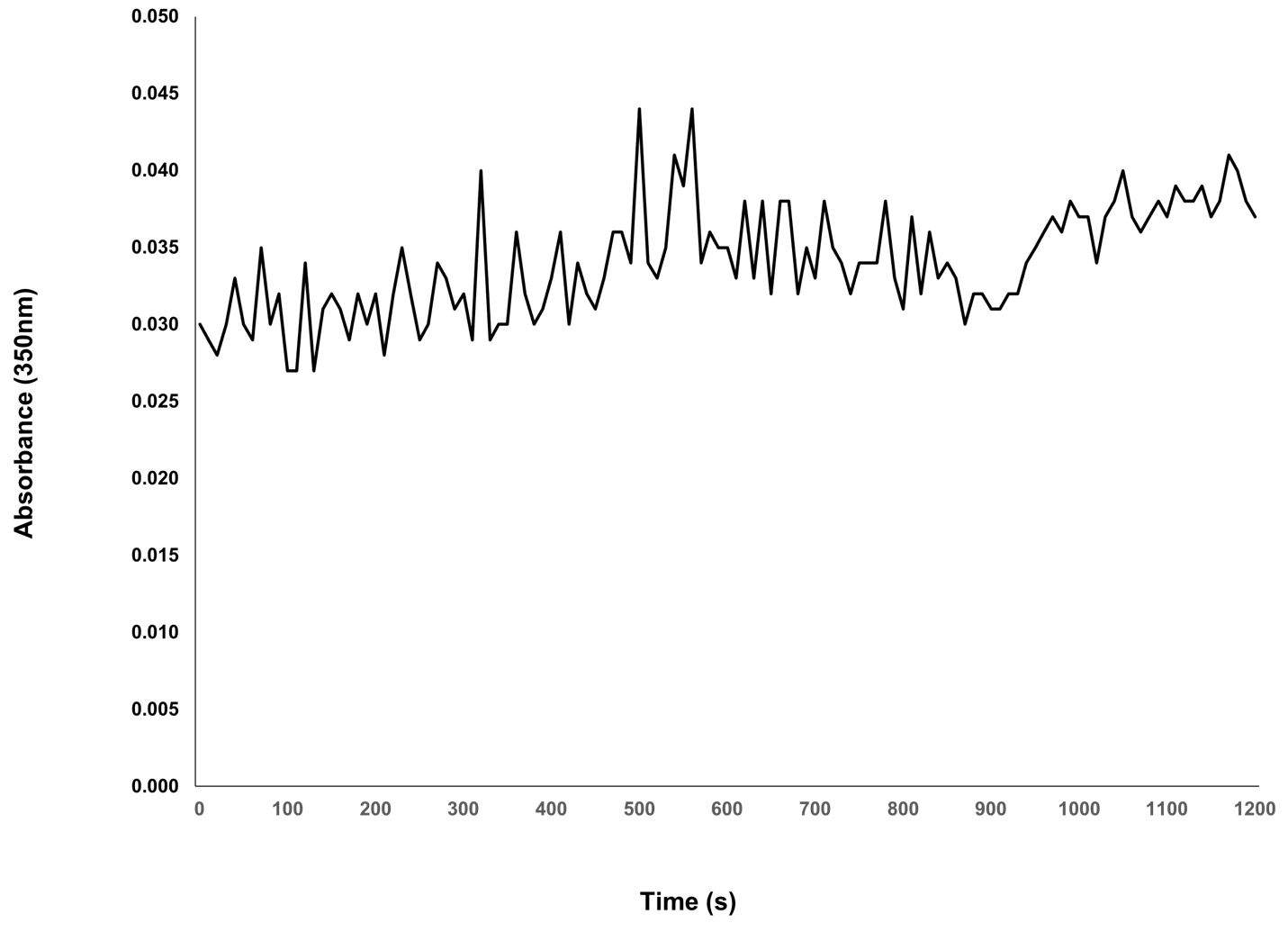


**Supplementary Figure 2**. The effect analysis of GppNHp on the assembly of the IRGB10 oligomer. Turbidity change of solution containing nucleotide-free IRGB10 was measured upon addition of GppNHp/MgCl_2_.


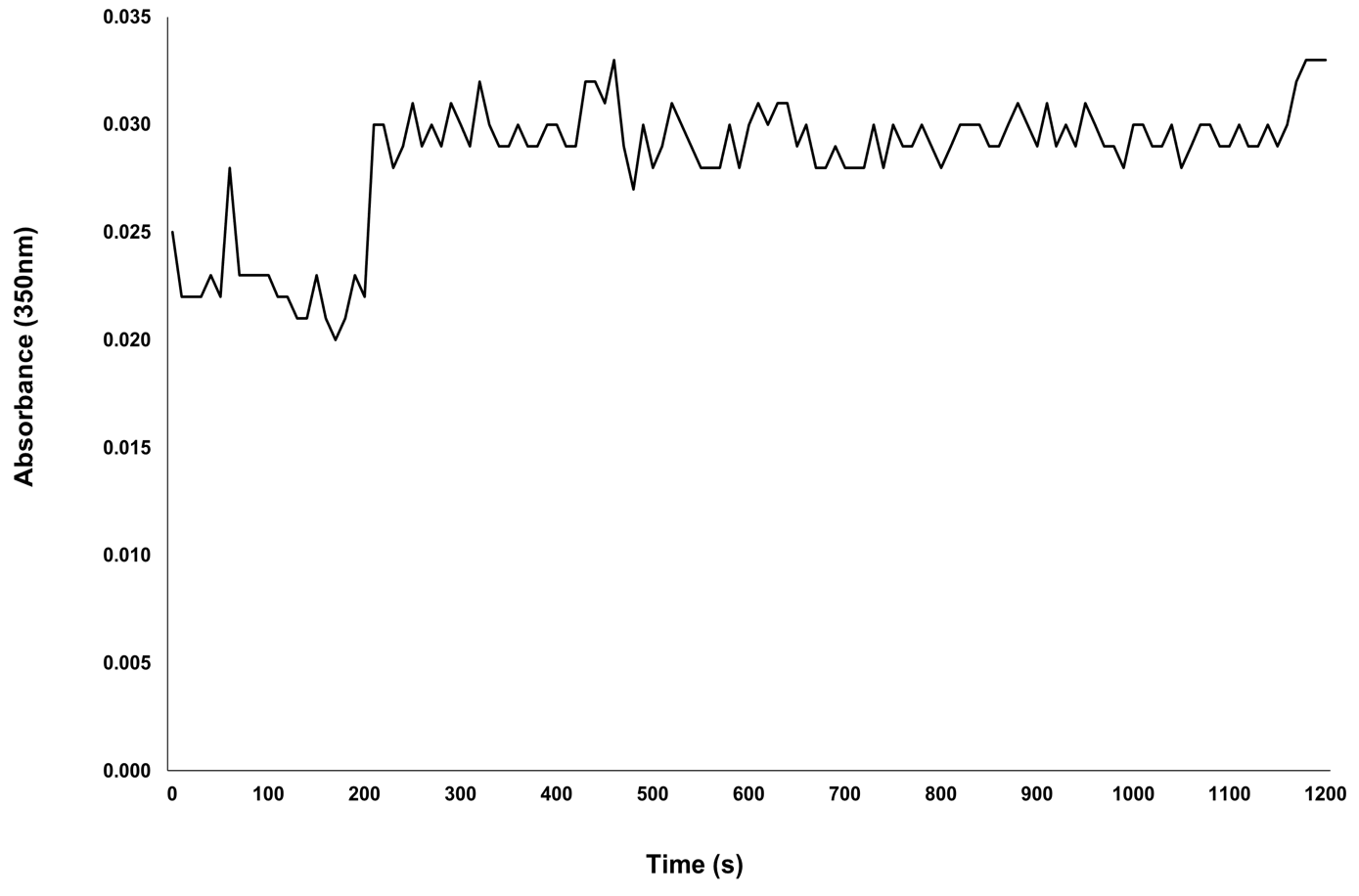


**Supplementary Figure 3**. The effect analysis of GDP on the assembly of the IRGB10 oligomer. Turbidity change of solution containing nucleotide-free IRGB10 was measured upon addition of GDP/MgCl_2_.


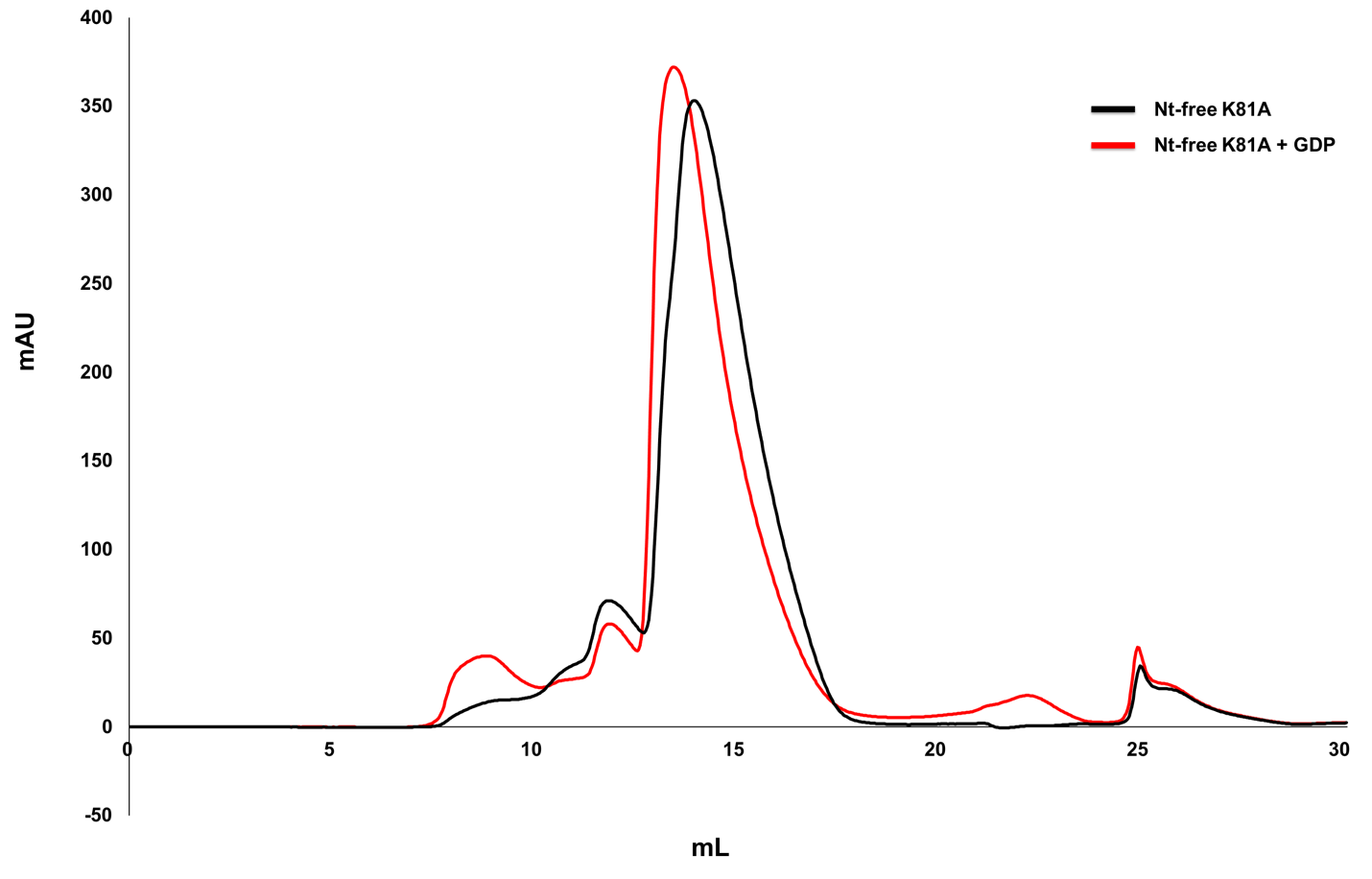


**Supplementary Figure 4**. SEC profiles of nucleotide-free (Nt-free) K81A (black line) and GDP-added K81A (red line).


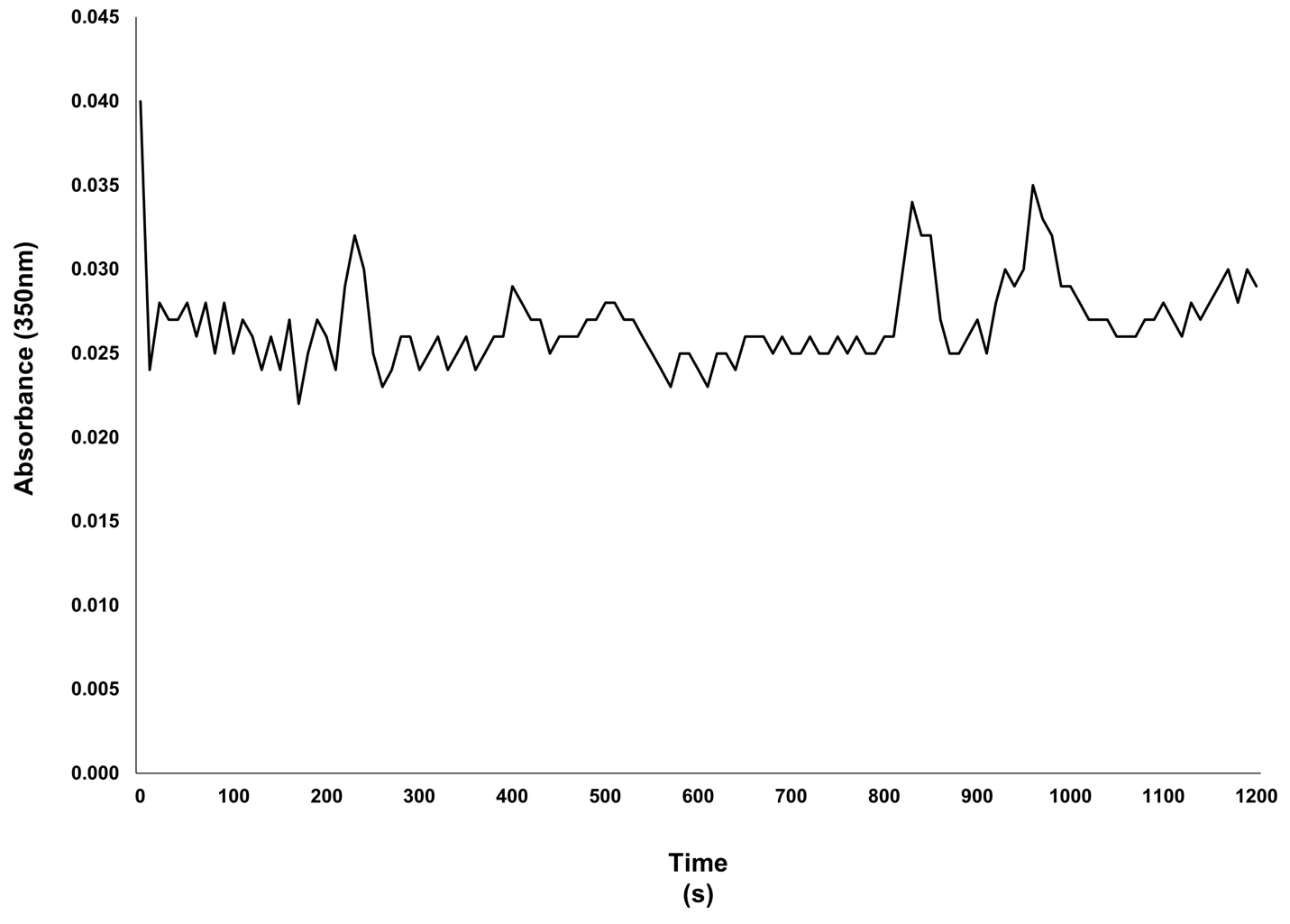


**Supplementary Figure 5**. The effect analysis of dimer disruption on the assembly of the IRGB10 oligomer. Turbidity change of solution containing nucleotide-free D185R mutant was measured upon addition of GTP/MgCl_2_.


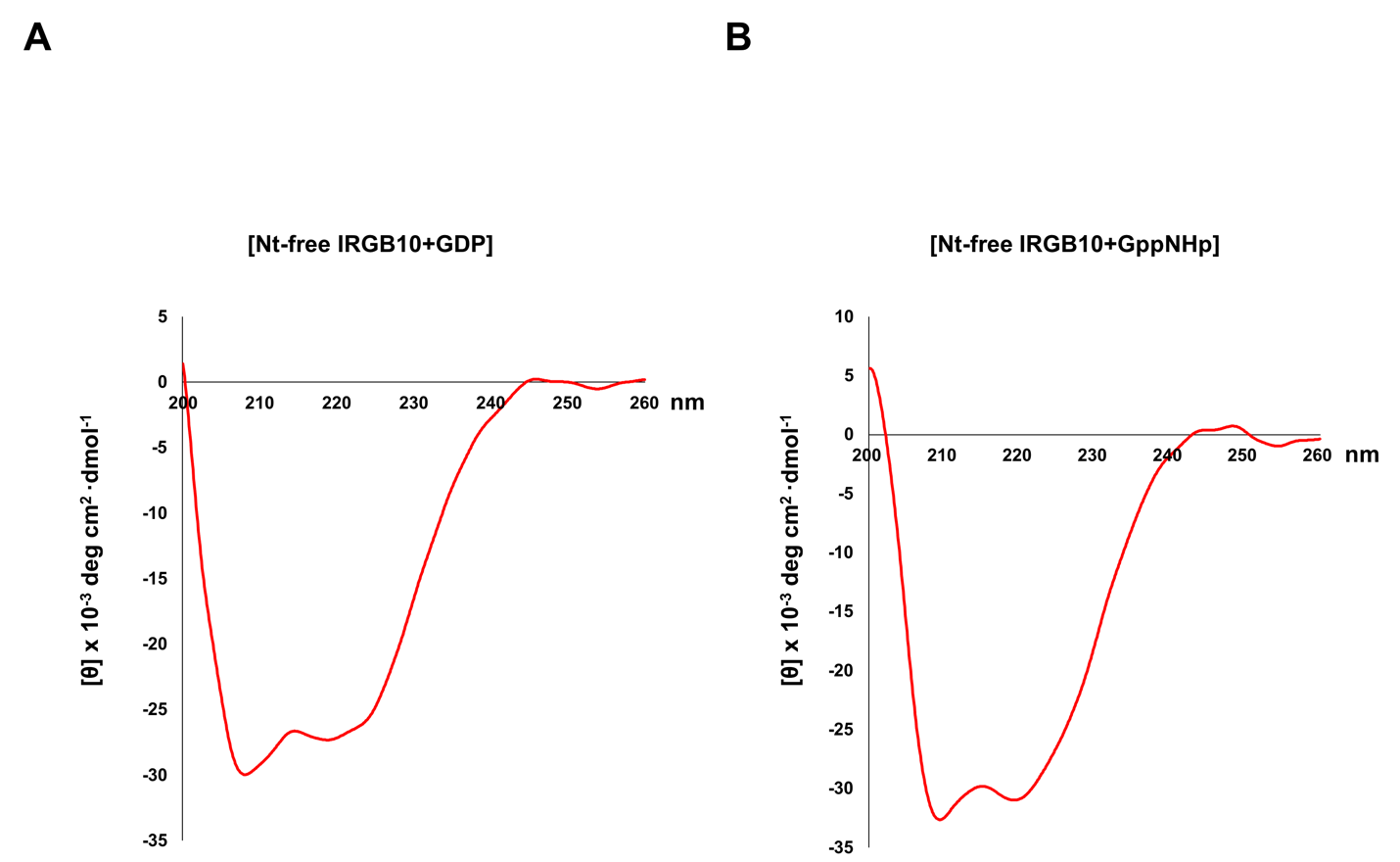


**Supplementary Figure 6**. Circular dichroic spectra of nucleotide-free (Nt-free) IRGB10 provided GDP (A) and provided GppNHp (B).
